# Clinical audit of psychotropic medication use at a South African intellectual disability hospital

**DOI:** 10.4102/sajpsychiatry.v32i0.2553

**Published:** 2026-01-21

**Authors:** Idorenyin U. Akpabio, Peter Smith, Sharon Kleintjes

**Affiliations:** 1 Department of Psychiatry and Mental Health, Faculty of Health Sciences, University of Cape Town, Cape Town, South Africa; 2 Department of Psychiatry, Western Cape Department of Health, Cape Town, South Africa

**Keywords:** intellectual disability, psychotropic prescribing, clinical audit, challenging behaviour, psychiatric care, guideline adherence

## Abstract

**Background:**

Individuals with intellectual disability (ID) are often prescribed psychotropic medications at disproportionately high rates, particularly for managing psychiatric symptoms or behaviours that challenge (BTC). International guidelines emphasise cautious, evidence-based use and the prioritisation of non-pharmacological interventions.

**Aim:**

This audit evaluated prescribing practices at a specialist psychiatric hospital in South Africa to determine the extent of alignment with internationally recommended standards for psychotropic use in individuals with ID.

**Setting:**

Outpatient department (OPD) of an advanced psychiatric care institution in Cape Town.

**Methods:**

A retrospective folder and prescription review was conducted for 103 patients with ID who were newly referred between January 2018 and August 2019. Prescribing decisions at the first visit and 6-month follow-up were assessed against guidance from the World Psychiatric Association (WPA) and National Institute for Health and Care Excellence (NICE).

**Results:**

Psychotropic medications were prescribed to 88% of patients. Antipsychotics were the most frequently used agents, comprising over half of all prescriptions, often for BTC in the absence of a psychotic disorder. While certain elements of guideline-based care were evident – such as use of low effective doses – gaps were noted in documentation of rationale, review scheduling, side-effect monitoring and consistent use of behavioural strategies. These areas highlight opportunities for strengthening practice.

**Conclusion:**

This audit emphasises the complexity of psychotropic prescribing for individuals with ID and the need for structured, multidisciplinary approaches to ensure safe and appropriate medication use.

**Contribution:**

Embedding standard protocols and regular reviews into clinical workflows may support better adherence to international best-practice standards.

## Introduction

Intellectual disability (ID), characterised by significant limitations in cognitive functioning and adaptive skills before age 18, affects approximately 1% of the global population.^1,2^ In South Africa, limited data suggest a higher prevalence, potentially because of the preventable conditions such as malnutrition, chronic infections (e.g. human immunodeficiency virus [HIV] and/or acquired immunodeficiency syndrome [AIDS], tuberculous meningitis), foetal alcohol-related syndromes and violence-related injuries.^3,4^ People with ID (PWID) are a vulnerable population, with a 2.5-fold increased risk of mental illness and a higher likelihood of medical comorbidities and behaviours that challenge (BTC), such as aggression, self-injury and temper tantrums.^5,6,7^

Several factors contribute to the complexity of diagnosing and managing mental illness in PWID. First of all, the predominance of medical, including specialist psychiatric training being centred on typically developing individuals, is a key factor in limited clinician confidence in engaging with, and delivering optimal care to PWID.^8,9,10^ Diagnosing mental illness in this population is further complicated by the severity of intellectual disability, atypical symptom presentation, communication difficulties, limited health literacy and barriers to accessing services.^6,11,12^ Behaviours that challenge may reflect underlying issues including pain, poor emotional regulation, social skill deficits or multimorbidity.^11,12,13,14,15^ These complexities often lead to psychotropic medications being overused or underused, and in some cases, prescribed without adequately investigating their underlying causes.^2,11,12,13,16,17,18,19,20^

Antipsychotics, which account for 30% – 50% of psychotropic prescriptions in PWID, are frequently used for BTC rather than psychosis, despite limited evidence of efficacy and risks of adverse effects like weight gain and extrapyramidal symptoms.^2,11,13,21^ These risks are heightened in PWID because of their reduced ability to report side effects, which may present as BTC and lead to diagnostic overshadowing.^14,22,23,24,25^ A 2010 Dutch study found that 58% of antipsychotic prescriptions were for behavioural issues, with only 25% for psychosis, while a 2015 review noted short-term efficacy of antipsychotics for BTC in children but cautioned against long-term use because of side effects.^11,13,21^ Psychotropic polypharmacy is also common, particularly among elderly PWID or those in residential settings.^24,26,27^

International guidelines, such as those from the National Institute for Health and Care Excellence (NICE) and the World Psychiatric Association (WPA), emphasise comprehensive assessment before prescribing psychotropics to PWID.^7,12,20,28,29,30,31,32^ However, no South African studies have examined psychotropic prescribing patterns in this population, and local guidelines are absent. This gap is critical given South Africa’s unique socio-economic and health challenges, which may influence ID prevalence and treatment practices.

This study addresses this gap by auditing psychotropic prescribing patterns among outpatients with ID at a specialist psychiatric hospital in South Africa. We aim to: (1) describe current prescribing practices, (2) identify factors associated with psychotropic medication use, and (3) evaluate adherence to international prescribing standards. By providing the first local evidence on this topic, the study seeks to inform best practices and improve care for PWID in South Africa.

## Research methods and design

This retrospective study reviewed patient folders and prescription charts of outpatients attending the outpatient department (OPD) of an advanced psychiatric institution in the Western Cape, South Africa, with initial consultation occurring between January 2018 and August 2019. The study period was selected to meet a minimum sample size of 72 files, identified following input from a statistician, based on the OPD’s patient volume. A total of 103 files were included at the initial visit, with 87 available at the 6-month follow-up. No exclusion criteria were applied; files of patients discharged or lost to follow-up by 6 months were analysed at initial assessment only.

The method and analysis of this study have been reported elsewhere as part of the author’s master’s thesis at the University of Cape Town.^33^

### Study setting

The research was carried out at an advanced psychiatric care institution in the Western Cape, South Africa, that provides inpatient services for adults and outpatient services for children, adolescents and adults with ID and complex mental health needs.^5^ Newly referred outpatients are screened at weekly multidisciplinary team meetings, where they may present with a confirmed or suspected ID diagnosis, with or without psychiatric comorbidities or existing medication.^33^ Prescribing and optimising medication are managed by a psychiatry consultant, rotating general psychiatry registrar and medical officer, using DSM-5 diagnostic criteria.

### Data collection

Data were extracted from folders at the initial visit and 6-month follow-up to assess prescribing practices over time.^33^ A data extraction form, developed based on WPA and NICE guidelines,^7,12,20,28^ captured demographic data (age, sex, living setting), clinical data (DSM-5 diagnoses, ID severity), and prescription details (medication type, dosage, indication). No distinction was made between medications initiated by referral facilities or the hospital. The lowest effective dose was determined by comparing prescribed dosages to recommended ranges. A trial run with 10 non-study folders refined the form, and a random subset of files was re-reviewed for accuracy. Data elements are summarised in Table 1 and Table 2, both reproduced from Akpabio et al.^33^

**TABLE 1:** Data extraction form: patient, diagnosis, and psychotropic prescription.

Variables	Category
1. Demographics	Age, sex, living arrangements (family home or residential facility)
2. Severity of ID	Mild, moderate, severe, profound, not documented
3. Patient diagnosis	Psychiatric: Behaviours that challenge, mood disorders (depressive and bipolar disorders), anxiety disorders, psychotic disorders, autism spectrum disorders (ASD), attention deficit hyperactivity disorder (ADHD), and other Medical: Epilepsy, dementia, and other
4. Psychotropic drug prescription	Grouped as antipsychotic agents, antidepressants, mood stabilisers, ADHD agents, anxiolytic or sedative agents, no medication, and other
5. Type of antipsychotic prescribed	Haloperidol, olanzapine, risperidone, and other

*Source*: Akpabio IU. A review of psychotropic drug prescription for patients with intellectual disability at Alexandra Hospital (a specialist Intellectual Disability psychiatric hospital) outpatient clinic [homepage on the Internet]. Faculty of Health Sciences, Department of Psychiatry and Mental Health; 2021 [cited 2025 May 25]. Available from: http://hdl.handle.net/11427/35575.^33^

ID, intellectual disability.

**TABLE 2:** Data extraction form based on World Psychiatric Association & National Institute for Health and Care Excellence guidelines.

Guideline-related variables
Rationale for antipsychotic use noted. Medication review schedule in the file. A standardised instrument for monitoring side effects in the file. Whether behavioural strategies were recommended. Whether support (e.g. referral to social work or other support groups/health services) was provided. Whether the lowest effective dose of the chosen psychotropic agent was prescribed.

*Source*: Akpabio IU. A review of psychotropic drug prescription for patients with intellectual disability at Alexandra Hospital (a specialist Intellectual Disability psychiatric hospital) outpatient clinic [homepage on the Internet]. Faculty of Health Sciences, Department of Psychiatry and Mental Health; 2021 [cited 2025 May 25]. Available from: http://hdl.handle.net/11427/35575.^33^

### Statistical analysis

Data were analysed using IBM SPSS Statistics for Windows, version 2025 (IBM Corp., Armonk, NY, US).^34^ Descriptive statistics included means, standard deviations (s.d.) and 95% confidence intervals for continuous variables, and frequencies and proportions for categorical variables. Chi-square tests examined associations between demographic (age, sex, living setting) and clinical factors (diagnosis, ID severity) and psychotropic prescriptions. Statistical significance was set at α = 0.05.^33^

### Ethical considerations

Ethical clearance to conduct this study was obtained from the Human Research Ethics Committee (HREC) of the Faculty of Health Sciences at the University of Cape Town (No. 809/2019). As the study involved a retrospective folder review without direct patient involvement, informed consent was not required. Clearance was obtained from the Western Cape Department of Health and the hospital’s Chief Executive Officer. Patient folders were assigned study numbers to ensure confidentiality.

## Results

The results presented here are based on findings originally reported in the author’s master’s thesis (Akpabio et al).^33^ All figures have been reproduced from the thesis.

### Demographic characteristics

Of the 103 outpatients, 58.2% (*n* = 60) were ≤ 18 years (30 children ≤ 12 years, 30 adolescents 13–18 years), 39.8% (*n* = 41) were 19–59 years, and 1.9% (*n* = 2) were ≥ 60 years. Male patients comprised 70% (*n* =72), with 64% (*n* = 46) of male patients and 45% (*n* = 14) of female patients ≤ 18 years. Most patients (87.4%, *n* = 90) lived with family; 13% (*n* = 13) resided in residential facilities, with only 2 (15%) of these ≤ 18 years.

### Intellectual disability and diagnoses

**FIGURE 1:**
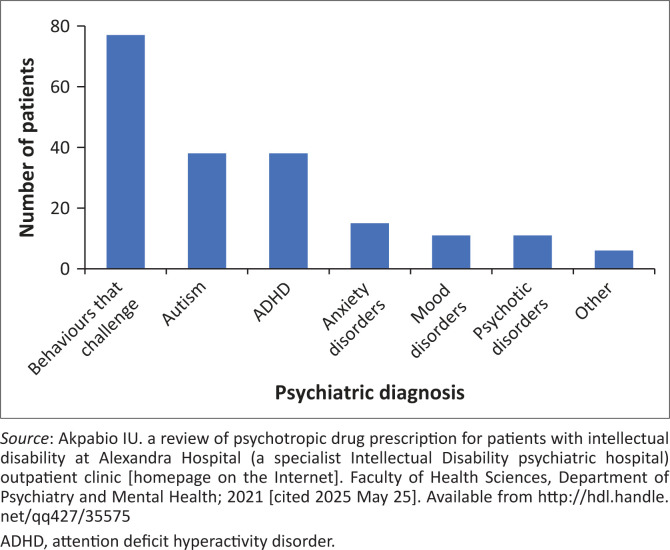
Psychiatric diagnoses of patients.

ID severity was mild in 26.2% (*n* = 27), moderate in 32% (*n* = 33), severe in 24.2% (*n* = 25), and profound in 1% (*n* = 1); 16.5% (*n* = 17) lacked documented severity. Psychiatric diagnoses (mean 1.9 per patient, s.d. = 0.8, range 1–4) included BTC in 74.8% (*n* = 77), autism spectrum disorder (ASD) or ADHD in 36.9% each (*n* = 38 each), psychotic disorders in 10.7% (*n* = 11), anxiety disorders in 14.6% (*n* = 15), and mood disorders in 10.7% (*n* = 11; 1 bipolar, 10 depressive). Children and adolescents accounted for 95% (*n* = 36) of ADHD, 62% (*n* = 48) of BTC, and 71% (*n* = 27) of ASD diagnoses, while adults comprised 91% (*n* = 10) of psychotic disorders, 44% (*n* = 7) of anxiety, and 100% (*n* = 10) of depressive disorders. The most common comorbid psychiatric diagnoses in patients with BTC (*n* = 45) were autism and ADHD, either as a dual or triple diagnosis. Comorbid medical conditions were present in 46% (*n* = 47), with epilepsy (*n* = 25) being most common. See Figure 1.

### Medication prescribing patterns

**FIGURE 2:**
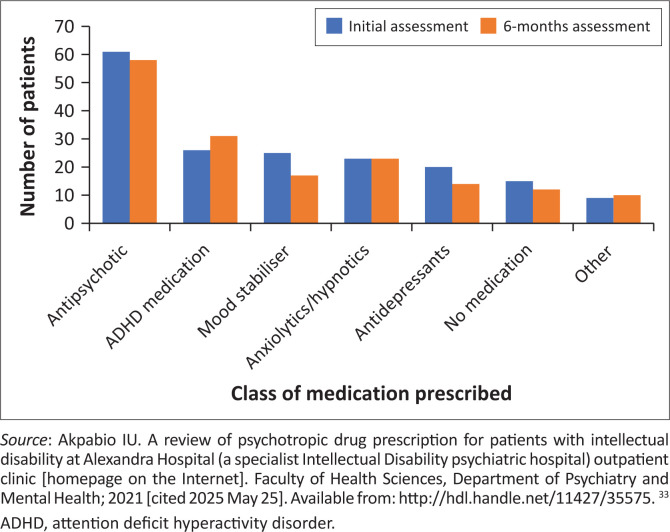
Medication prescribed at initial and 6-month assessment.

**FIGURE 3:**
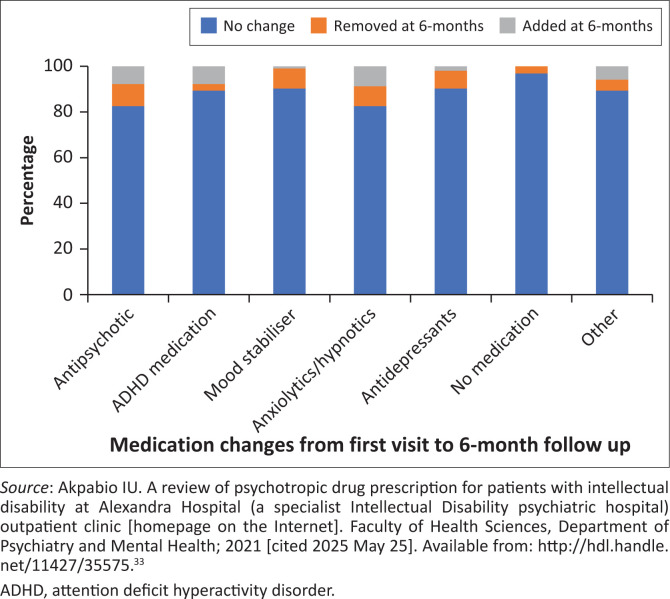
Medication changes.

Antipsychotics were prescribed to 59.2% (*n* = 61) of patients at initial assessment and 56.3% (*n* = 49) at 6-month follow-up, with risperidone and olanzapine being the most common medications (Figure 2). Children and adolescents received antipsychotics more frequently (56% initial, 62% follow-up) than adults. Anticonvulsant mood stabilisers were primarily used for epilepsy, but were used to manage BTC in 33% (*n* = 8) of patients initially and 24% (*n* = 4) at follow-up. The mean number of psychotropics was 1.61 (s.d. = 1.1) initially and 1.65 (s.d. = 1.1) at follow-up. At initial assessment, 14.6% (*n* = 15) received no medication, 35% (*n* = 36) one, 32% (*n* = 33) two, and 18.4% (*n* = 19) three or more. At follow-up (*n* = 87), 13.8% (*n* = 12) received no medication, 27.6% (*n* = 24) one, 27.6% (*n* = 24) two, and 31% (*n* = 27) three or more (Figure 3). At both visits, children and adolescents were more commonly prescribed two or fewer medications, whereas adults were more likely to be treated with three or more psychotropic agents. Over 80% of patients had no medication changes between visits.

### Associations between prescribed medications, demographics and diagnoses

Patients ≤ 18 years were significantly more likely to receive ADHD medication (initial: 41.7%, *n* = 25/60 vs. 2.3%, *n* = 1/43, *p* < 0.001, Cramer’s *V* = 0.45; follow-up: 50%, *n* = 30/60 vs. 2.3%, *n* = 1/43, *p* < 0.001, Cramer’s *V* = 0.51). Adults (≥ 19 years) were more likely to receive anxiolytics and/or hypnotics (32.6%, *n* = 14/43 vs. 15%, *n* = 9/60, *p* = 0.035, Cramer’s *V* = 0.21) and antidepressants (37.2%, *n* = 16/43 vs. 6.7%, *n* = 4/60, *p* < 0.001, Cramer’s *V* = 0.38) at initial assessment. Female patients were more likely to receive anxiolytics and/or hypnotics (35.5%, *n* = 11/31 vs. 16.7%, *n* = 12/72, *p* = 0.065, Cramer’s *V* = 0.18). Patients in residential facilities were more likely to receive anxiolytics/hypnotics (53.8%, *n* = 7/13 vs. 16.7%, *n* = 12/72, *p* = 0.007, Cramer’s *V* = 0.28). ID severity was not associated with medication type. Patients with ADHD (63.2%, *n* = 24/38 vs. 3.1%, *n* = 2/65, *p* < 0.001, Cramer’s *V* = 0.67) and mood disorders (63.6%, *n* = 7/11 vs. 12%, *n* = 11/92, *p* < 0.001, Cramer’s *V* = 0.55) were more likely to receive ADHD medication and antidepressants, respectively, at initial assessment.^33^

### Compliance with prescribing guidelines

A rationale for antipsychotic use was documented in 68.9% (*n* = 42/61) of cases, primarily for BTC (57.1%, *n* = 24). All antipsychotic prescriptions used the lowest effective dose. However, no files included review schedules or standardised side-effect monitoring. For BTC (*n* = 77), 46.8% (*n* = 36) were managed with behavioural interventions alone initially, with nine later receiving medication by 6 months. The remainder received medication alone or combined with behavioural interventions. All patients were informed of support structures (e.g. Autism Western Cape, social services) and referred to psychology or occupational therapy when appropriate.

## Discussion

This retrospective study audited psychotropic prescribing patterns among 103 outpatients with ID at an advanced psychiatric care institution in the Western Cape, South Africa.^33^ The aim was to assess adherence to international guidelines, including those from the WPA and the NICE.^7,12,20,28,33^ Approximately 88% (*n* = 91) of patients received at least one psychotropic agent, a figure that aligns with global trends of high psychotropic use among people with intellectual disability (PWID).^2,11,28^

Prescribing patterns generally reflected clinical diagnoses: stimulants were used for ADHD, and antidepressants for anxiety or mood disorders.^7,16,35^ Antipsychotics, however, were the most commonly prescribed class – 59.2% at baseline and 56.3% at 6 months – despite only 10.7% of patients being diagnosed with a psychotic disorder. This mirrors international findings of frequent off-label antipsychotic use to manage BTC, rather than psychosis.^2,11,13,25^ Among those prescribed antipsychotics, risperidone was the agent of choice, particularly for children, adolescents and individuals with ASD, and was commonly used in the context of BTC. Encouragingly, it was prescribed at the lowest effective dose, with rationale documented in nearly 69% of cases. However, only 46.8% of BTC cases were initially managed with behavioural strategies alone – despite strong WPA and NICE recommendations to prioritise non-pharmacological interventions.^2,6,11,36,37^

Age and living arrangements appeared to shape prescribing trends. Children and adolescents (≤ 18 years) were more likely to receive ADHD medications, while adults (≥ 19 years) were prescribed more anxiolytics, hypnotics and antidepressants – consistent with developmental trajectories of psychiatric diagnoses. Female patients and those in residential facilities were more likely to receive anxiolytics or hypnotics, consistent with evidence linking institutional living to increased exposure to psychotropic medication and polypharmacy.^23,26,28^ Notably, medication choices were not influenced by ID severity, which aligns with prevailing guidelines that do not stratify treatment by severity level.^7,12^

Despite alignment in many areas, notable gaps emerged in guideline implementation. No reviewed files included standardised review schedules or systematic side-effect monitoring, which are especially critical for PWID given their increased vulnerability to adverse drug effects.^25^ More than 80% of patients remained on the same medications over a 6-month period, raising concerns about inadequate review of efficacy – particularly in cases involving antipsychotics for BTC, where guidelines explicitly caution against prolonged use without regular reassessment.^12,15,18^

Several systemic improvements could enhance adherence to best-practice care. For example, standardised templates for documenting treatment rationale, setting review schedules and recording adverse effects would support clinicians – especially in pressured outpatient settings – in delivering more consistent, guideline-concordant care.^12,21,29,30,31,32^ Additionally, targeted clinician training on ID-specific care could improve confidence in using behavioural approaches and reduce over-reliance on pharmacologic strategies.^38^ Given South Africa’s unique socio-economic context, there is an urgent need to develop and test locally appropriate behavioural interventions for managing BTC.^39^

### Strengths and limitations

This is the first known study to examine psychotropic prescribing practices for PWID in South Africa, providing a crucial baseline for evaluating and improving care at the study site. Its findings offer practical insights that may inform clinical protocols, training needs and policy at both local and national levels. However, the retrospective folder review design introduces limitations because of the variable completeness of documentation. It is unclear whether some prescribing decisions were discussed but not recorded. Moreover, although clinicians at the facility use DSM-5 diagnostic criteria, the fidelity of diagnosis was not independently assessed, leaving room for misclassification. Finally, as a single-site study, findings may not be generalisable to other outpatient settings.

## Conclusion

This study provides a valuable initial glimpse into psychotropic prescribing practices for individuals with ID in a South African outpatient psychiatric setting. While prescribing largely aligned with clinical indications for ADHD, anxiety and mood disorders, antipsychotic use was widespread – often for BTC rather than psychosis – and rarely accompanied by behavioural interventions alone. Clinicians demonstrated caution around polypharmacy and prescribed at the lowest effective doses, but gaps remain in review scheduling, rationale documentation and side-effect monitoring. Strengthening clinician training, adopting standardised documentation tools and developing context-specific behavioural interventions are critical next steps. These findings offer a foundation for improving practice at the study site and suggest the need for broader audits and implementation strategies across the country.
